# Network Analysis of Functional Genomics Data: Application to Avian Sex-Biased Gene Expression

**DOI:** 10.1100/2012/130491

**Published:** 2012-12-23

**Authors:** Oliver Frings, Judith E. Mank, Andrey Alexeyenko, Erik L. L. Sonnhammer

**Affiliations:** ^1^Stockholm Bioinformatics Centre, Science for Life Laboratory, Box 1031, SE-171 21 Solna, Sweden; ^2^Department of Biochemistry and Biophysics, Stockholm University, SE-106 91 Stockholm, Sweden; ^3^Department of Genetics, Evolution and the Environment, University College London, WC1E 6BT, UK; ^4^School of Biotechnology, Royal Institute of Technology, SE-171 65 Solna, Sweden; ^5^Swedish eScience Research Center, SE-100 44 Stockholm, Sweden

## Abstract

Gene expression analysis is often used to investigate the molecular and functional underpinnings of a phenotype. However, differential expression of individual genes is limited in that it does not consider how the genes interact with each other in networks. To address this shortcoming we propose a number of network-based analyses that give additional functional insights into the studied process. These were applied to a dataset of sex-specific gene expression in the chicken gonad and brain at different developmental stages. We first constructed a global chicken interaction network. Combining the network with the expression data showed that most sex-biased genes tend to have lower network connectivity, that is, act within local network environments, although some interesting exceptions were found. Genes of the same sex bias were generally more strongly connected with each other than expected. We further studied the fates of duplicated sex-biased genes and found that there is a significant trend to keep the same pattern of sex bias after duplication. We also identified sex-biased modules in the network, which reveal pathways or complexes involved in sex-specific processes. Altogether, this work integrates evolutionary genomics with systems biology in a novel way, offering new insights into the modular nature of sex-biased genes.

## 1. Introduction

Although primary sex determining genes are responsible for the initial sex determining cues in the gonad, most of the heritable differences in morphology, behavior, and life history between males and females are the result of different expression levels of genes present in both sexes [1, 2]. Sex-biased genes, which comprise up to 50% of metazoan transcriptomes [3–7], are the product of sexually antagonistic selection for different male and female optima [8, 9]. This antagonism is resolved with the emergence of sex-specific transcriptional regulatory elements that decouple expression between the sexes, thereby allowing separate female and male phenotypes to emerge from a shared genome. Sex-biased genes behave according to the evolutionary predictions for sexually selected and sexually antagonistic traits [10–18], and the study of sex-biased gene expression is emerging as a method to connect sex-specific selection pressures, which act on the whole organism, to the encoding loci.

This connection between sex-biased genes and sexually dimorphic traits offers a way to study the complex interactions between the phenotype to the underlying genome. Most studies of sex-biased gene expression treat individual genes as independent units, ignoring correlated expression that results from the interactive nature of genetic pathways and networks. This simplification compresses the multidimensional nature of the transcriptome. However, because many sexually dimorphic phenotypes are complex amalgams of numerous genes [5, 19, 20], we require a way to study the interactions of the genes underlying them if we wish to understand the constraints acting on these traits and how they respond to selection. In addition to this complexity, many genes contribute to more than one phenotype, pathway, or subnetwork. This pleiotropy is likely an important factor in the evolution of sex-biased gene expression which may ameliorate intralocus sexual conflict acting on a given gene [9].

For genes with high levels of pleiotropy, the many functions of a single locus result in strong evolutionary constraints hindering change due to selection pressure for any single function [21]. This is important for studies of sex-biased genes, as sex-biased gene expression patterns resulting from sexually antagonistic selection for any single function may be detrimental in other functionalities [22]. This would suggest that genes with many pathway connections, though not necessarily less likely to experience sexually antagonistic selection, are less likely to resolve that antagonism through sex-biased expression, as this could result in detrimental effects in other phenotypes encoded by the same loci [23]. More simply stated, the resolution of sexually antagonistic selection may be more common for genes with fewer network interactions. This prediction suggests that (1) pleiotropic genes may contain relatively high levels of unresolved sexual conflict and (2) sexually dimorphic phenotypes are more often encoded by genes with few other functions. This has important implications for evolutionary models of sexual selection which typically assume single functionalities and simple inheritance patterns.

Here we test the relationship between network interaction and sex-biased gene expression with a newly developed gene interaction atlas of the chicken. Previously, we have shown that sex-biased expression is prevalent in chicken [23] and that sex-biased genes in chicken exhibit evolutionary patterns consistent with sexual selection and sexual conflict [16, 18, 24]. In this analysis, we created a functional coupling network from data integration [25] of chicken and incorporated sex-biased expression data into it in order to analyze the connectivity of sex-biased and unbiased genes in both the gonad and soma. Overall, our goal was to better understand the relationship between sexually dimorphic phenotypes, the sexually antagonistic selection pressures shaping them, and the genes encoding them.

## 2. Materials and Methods

### 2.1. Network

The chicken network was generated using the FunCoup framework [25, 26]. This framework reconstructs global large-scale networks of functional coupling by Bayesian integration of diverse high-throughput data-sets. More specifically raw scores of various types of functional coupling are turned into probabilistic estimates that are then integrated across different types of data and model organisms. The different types of evidence comprised: protein-protein interactions, mRNA coexpression, subcellular colocalization, phylogenetic profile similarity, cotargeting by either miRNA or transcription factors, protein co-expression, and domain-domain interactions. The integration of data from different sources enabled more comprehensive network reconstruction with higher quality. Furthermore, data from other eukaryotic species were transferred via orthologs. Ortholog assignments for cross-species mapping were obtained from the InParanoid database [27]. Signaling and metabolic pathways from KEGG as well as both pathway types combined were used as gold standard for Bayesian training. The network has consequently three different kinds of links: metabolic, signaling, and combined. The network was predicted using seven chicken-specific microarray expression datasets (see Table S1 in Supplementary Materials available online at doi:10.1100/2012/130491), phylogenetic profile similarity across eukaryotes, and information transferred from other species via orthologs. The use of ortholog transfer was of special importance in this case, as it allows us to overcome the lack of chicken-specific interaction data.

### 2.2. Microarray Expression Datasets

The network was studied in the context of sex bias under different conditions. We used three different Affymetrix chicken expression datasets from the embryonic gonad, the adult gonad, and the adult brain (previously described in Mank et al. [16], Mank and Ellegren [28], and Mank et al. [24]). Each tissue/time-point array hybridization was based on three replicate nonoverlapping within-sex pools of 3–5 individual samples from male and female embryonic and adult chickens. All datasets were normalized using the MAS5 algorithm from the Affy Bioconductor package.

### 2.3. Differential Gene Expression

There are several different ways to define differential gene expression. Traditionally genes that are meant to be over- or underrepresented in one condition compared to a second condition have been identified by fold-change. Although this method is still widely used, it might be biased in multiple ways. A high fold-change can be caused by a single flawed sample or by negligible differences in expression level just above the detection limit. In other words it ignores if the differences in expression change are statistically significant or not. Different methods have been proposed to assess the significance of changes in gene expression. The Student's *t*-test and Welch test are commonly used to estimate the significance of differential gene expression. However, the reliability of those methods strongly depends on the sample variance and the number of samples for each condition. Besides numerous statistical packages have been developed that account for differential gene expression, for example, SAM, EBAM, and so forth.

It also has been widely recognized that using different methods might result in rather distinct sets of differential expressed genes. We approached the problem by using the R MWT-package to determine significant differential gene expression [29]. The MWT method is essentially a moderated Welch test that aims to circumvent the problem of a low sample number by pooling the variance over the whole probe set. To adjust for multiple testing, all *P* values related to differential expression were corrected using the Benjamini-Hochberg method [30] that is rendered into false discovery rate (FDR) values.

### 2.4. Network Randomization

To determine the significance of the level of connectivity between a predefined set of genes and a second set (or itself) we used the CrossTalkZ network randomization package (http://sonnhammer.sbc.su.se/download/software/CrossTalkZ/). The method compares the number of observed connections between two gene sets to the number of connections in a randomized version of the network. In the course of network randomization, links between genes are swapped so that the original connectivity of a gene is conserved. The randomization was repeated 100 times, and all results were averaged. For each gene set a number of statistics were calculated including a *z*-score, a *P* value, and a Benjamini-Hochberg corrected FDR value.

### 2.5. Functional Gene Modules

To identify gene modules that are relevant to different developmental stages and sexes we compiled for each condition networks of male or female-biased genes separately. In addition these networks contained other genes strongly connected to those sex-biased genes. We used the hypergeometric test to identify such genes, and genes with a Bonferroni corrected *P* value of less than 10% were included in such networks.

A large number of network clustering techniques exist to infer modules, but it is not obvious which ones are most robust, that is, perform well under many different circumstances. From a benchmark study of 8 popular methods we selected the two overall top performing methods, MGclus (http://sonnhammer.sbc.su.se/download/software/MGclus/) and MCL [31]. The latter was used with an inflation parameter of 3.5. The significance of the derived modules was evaluated by comparing the number of enriched GO terms per module to the expected number of enriched GO terms given a set of genes of that size. The expected number of GO terms per module was estimated by 500 times randomly picking *n* genes from the parental subnetwork, where *n* equals the number of genes for a module. Based on the distributions of the expected numbers of enriched GO terms, a *z*-score was calculated for enrichment of GO terms per clustering.

## 3. Results

### 3.1. The Chicken Network

With the FunCoup tool and dataset collection, we derived a global chicken gene interaction network. FunCoup can be used to determine confidence values regarding the value of observed functional coupling links, and the chicken network has roughly 1.8 million links at a confidence cutoff (*c*) > 0.02 and about 58,000 at *c* > 0.75 (Table 1). The network was trained on three different categories: metabolic, signaling, and both metabolic and signaling combined. In the following we used a *c* > 0.25 as it represents a reasonable tradeoff between accuracy and coverage.

The proteins with the highest connectivity are mainly related to fundamental cellular processes such as protein synthesis and degradation, translation, and transcription (Table S2). Many of them are involved in multiple processes. The most connected protein in our chicken network is the RA-related nuclear protein (RAN). Due to its various functions in nuclear transport and cell cycle regulation, it acts as a major hub with a host of other proteins. Interestingly, RAN is highly differentially expressed between male and female chicken (i.e., sex biased) in the gonad (FDR *P* < 10^−4^ in the adult), which is actually less common for hubs as we show in the following.

### 3.2. Sex Bias Depends on Network Connectivity

Is there a dependency between sex bias and network connectivity? To answer this question, we first grouped the genes in three sex bias categories: male biased, female biased, and unbiased. For this we used the MWT statistic of differential expression with an FDR *P* value cutoff of 0.1. This was done for all four tissue/stage conditions: the embryonic and adult gonad and brain. The number of sex-biased genes in the network for each category is shown in Table 2. Remarkably, the embryonic brain contained almost no sex-biased genes and was therefore left out of this analysis. The adult brain had more sex-biased genes, but these still represented only 3% of the genes in the network. In contrast, the gonad abounded with sex-biased genes in the network: 43% in the embryo and 82% in the adult.

Sex-biased hub genes were thus frequent in the gonad, but not in the brain, and this may be due to the fact that the male and female gonads have extensive sex-specific functions, while the brain consists of many different tissues of which only small fractions of our microarray samples may be affected by the sex. The sex-specific expression signal in the brain will therefore be diluted by the nonaffected tissues until it is no longer statistically significant. Finer-scale analysis of specific brain tissues might reveal more dimorphism in gene expression, particularly those regions related to vocalization differences between male and female birds [32] or reproductive behavior [33].

We calculated Spearman's rank correlation coefficient between FDR values from differential expression analysis and node degree (i.e., the number of connections a gene has in the network), for each tissue/stage combination. As can be seen in Table 3, all but one of the sex-biased categories in the gonads had a significant positive correlation at FDR *P* < 0.1, indicating a tendency for fewer network connections as sex-bias increased. The exception was male biased genes in the adult gonad, but when lowering the cutoff to 0.001 these gave a weak but significant positive correlation (*r* = 0.1, *P* < 0.05). Unbiased genes were not significantly correlated with connectivity, nor were the brain genes, as may be expected given the dilution problem of brain expression mentioned above. This trend also held true when using fold-change as a measure for sex-bias. In other words, sex biased pathways seemed to generally affect local components of the network, except for the ones overexpressed in the male adult gonad, which tends to act more often in global components.

### 3.3. Sex-Biased Hub Genes

From the previous section it is clear that the level of sex was a function of both by tissue/stage condition as well as connectivity. We demonstrated that low connectivity genes tend to be more sex biased than high connectivity genes, yet some hub genes have strong sex bias. To focus on such sex-biased hubs, we first ranked each gene according to sex bias or connectivity separately and then reranked them according to the sum of both ranks. The highest ranked genes thus represent the most sex-biased hub genes. Table S3 shows the twenty top ranked sex-biased hubs in each condition.

Among the highly connected hubs in Table S3, tubulin alpha-3e (TUBA3E), ranked first and second in the embryonic and adult gonads, has 426 links. It is female biased in the embryonic gonad but male biased in the adult gonad. Other highly connected tubulins are also in the list. This indicates that sexual differentiation and sex-specific function are partly orchestrated via sex-specific tubulin assembly. Some of the proteins in Table S3 are directly implicated in sex determination, for example, the testis-specific tubulin alpha-2 (TUBAL2, connectivity 94), the meiotic recombination SPO11 (connectivity 37), or the NASP the nuclear autoantigenic sperm protein (connectivity 97). A major hub in the embryonic as well as the adult gonad is CDK3, cell division protein kinase 3 (connectivity 189). CDK3 is further linked to the KEGG pathways oocyte meiosis as well as progesterone-mediated oocyte maturation. Intriguingly, CDK3 was strongly female biased in the embryonic gonad but strongly male biased in the adult gonad. Overall this suggests that a major difference between the sexes results from a complex interplay between components of the cell division and development systems.

### 3.4. Interconnectivity of Sex-Biased Genes

**Figure 1:**
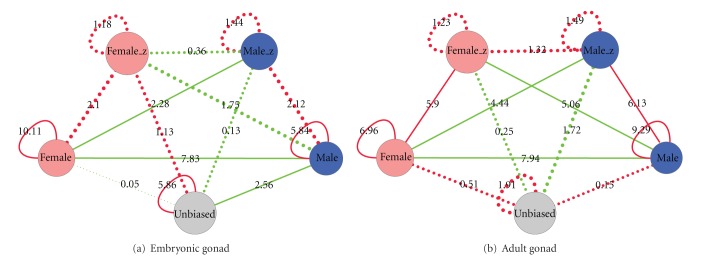
Network of crosstalk, that is, enrichment or depletion of links, between sex-biased and unbiased genes. Positive crosstalk (i.e., enrichment of links) is shown in red and depletion in green. Solid lines indicate significant crosstalk with FDR < 0.05. Edge width and label show the *z*-score of the crosstalk analysis.

To answer the question if sex-biased genes are stronger connected to genes of the same bias, we compared the connection frequencies between the different categories to what is expected by chance alone. For topology-preserving randomization of the network we used the CrossTalkZ program (see Section 2) and performed 100 randomization runs. The results for the embryonic and adult gonads are shown in Figure 1.

In the gonad we found genes of the same sex bias (e.g., male versus male) to be more frequently connected to each other than to genes of a different sex bias or unbiased genes. It is striking that both in the embryonic and adult gonads, male-biased genes have significantly fewer connections to female-biased genes than expected by chance. In the brain, we did not observe a significant crosstalk between male- or female-biased genes, probably due to the dilution problem mentioned above. Separate female- and male-specific networks are thus common throughout the chicken network in the gonad, and these sex-specific networks function to encode dimorphic processes in this tissue.

Sex-biased genes on the Z-chromosome are shown as separate nodes in Figure 1. The Z chromosome had to be treated separately from the autosomes due to the lack of complete dosage compensation in birds which results in a pervasive male bias for nondosage sensitive genes [34]. Sex-biased genes on the Z-chromosome are shown as separate nodes in Figure 1. Genes of the same sex bias located on the Z-chromosome were more connected to each other than expected by chance and were significantly enriched in links to genes of the same sex bias on other chromosomes. Connections between female and male genes on the Z-chromosome were about as frequent as expected, but there were significantly fewer connections than expected between whole-genome male and Z-chromosome female-biased genes and vice versa. These results show that the reconstructed chicken network is largely made up of male-specific and female-specific modules.

### 3.5. Duplicated Sex-Biased Genes

Gene duplication is a mechanism for creating new functions, and such a functional niche could be associated with a particular sex bias. Previous work has shown that duplicates of unbiased genes often develop sex-biased expression [35]. However, it is not yet clear if sex-biased genes that were recently duplicated tend to maintain the same pattern of expression bias. To answer this question, we restricted the analysis to orthologs. Orthologous genes are known to retain identical or closely related biological function more often than other types of homologs [36–39]. Two genes in one species are considered as inparalogs with respect to another species if the gene duplication occurred after the respective speciation event. In order to clarify if inparalog genes in chicken would more often have the same sex bias or are biased towards the opposite sex, we selected all inparalogs between chicken and human from the InParanoid database [27]. An ortholog group was only analyzed if it had at least two alternative inparalogs in chicken and if expression data were available. Roughly half of the groups could thus be analyzed. The group was then evaluated for differentially expressed genes using a FDR cutoff of 10%. 

**Figure 2:**
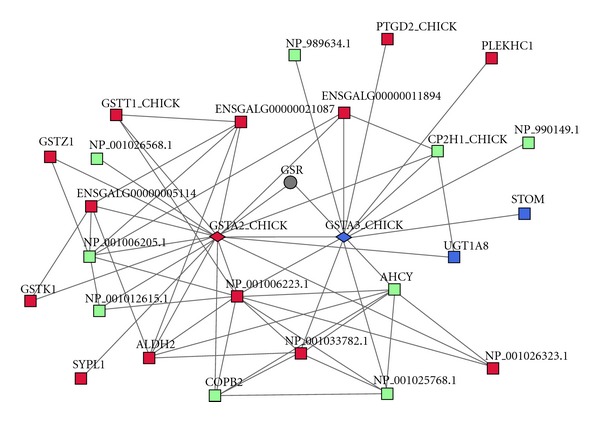
Example of sex-differentiation-driven subfunctionalization. The chicken genes GSTA2 and GSTA3 (glutathione S-transferases 2 and 3; shown as diamonds) originate from a duplication that happened after the divergence from human, making them inparalogs. GSTA2 is male biased, but GSTA3 is female biased in the adult gonad. Their interaction partners in the chicken network are shown with sex bias. Male-biased genes are shown in blue, female-biased in red, unbiased in green, and unknown in grey.

The results of this analysis can be seen in Table 4 (and Table S4). In the gonad, the numbers of male- and female-biased groups were similar, while in the brain none of the groups were biased towards one of the sexes. A big fraction of the groups is however a mix between sex-biased and unbiased genes. Remarkably, five ortholog groups in the adult gonad contained both male- and female-biased genes. An example of such a group contained female-biased glutathione S-transferase 2 (GSTA2) and male-biased glutathione S-transferase 3 (GSTA3). These inparalogs were connected to each other in the FunCoup network as well as to a set of other sex-biased genes (see Figure 2). However, 75% of GSTA2 links and 48% of GSTA3 links were not in common. At a cutoff of 0.5 only 2 links were shared between the two genes. It thus represents a likely example of subfunctionalization driven by sex differentiation.

To evaluate significance of these findings we compared the obtained numbers of inparalogs with the same bias to the distribution expected by chance (see Table S4). To this end, we randomly sampled genes of each ortholog group from the complete expression dataset. This procedure was repeated 1000 times, and the obtained numbers of groups with the same sex bias were compared to the observed values. For both the embryonic and adult gonadsthe original number of inparalogs with the same sex bias significantly exceeded the number of what would be expected by chance alone (*P* < 0.05). In the brain however there was no clear trend. We conclude that inparalogs that emerged after the mammal-bird speciation generally preserved sex bias, although a few exceptions exist.

### 3.6. Sex-Biased Network Modules

Network modules, or clusters, can be useful to find groups of functionally related genes. Such modules may represent parts of pathways or complexes that can be discerned as cliques of genes that are strongly linked to each other in the network. To identify functional modules of sex-biased genes, we calculated for each condition a set of male- or female-biased modules. We derived a network of sex-biased genes as well as genes which were strongly connected to them for each condition (see Section 2). Different clustering methods have different advantages and disadvantages and might as well result in relatively different sets of modules. We used two different methods to derive functional modules, MCL and MGclus. In the following we contrast the results of both methods as well as discuss the significance of the derived modules based on a few selected examples.

MCL is a global clustering approach that simulates random walks in the underlying interaction network. MGclus tries to identify clusters of strongly mutually linked genes using a scoring function that additionally accounts for shared neighbors. Thus nodes in the same cluster are thus likely to share a large fraction of shared neighbors, which increases cohesiveness within the cluster.

The overall outcomes of the MGclus and MCL clusterings are shown in Table 5. In all of the cases the clusters were significant, that is, had at least one enriched GO term, which was assigned to more than one gene in the cluster. Further, in all cases except for the adult brain, all clusters had on average significantly more enriched GO terms than random modules of the same size. The adult brain might however have too few sex-biased genes to see this. It is also worth noting that the MGclus clusters had on average more enriched GO terms than the MCL clusters.

How different are MCL and MGclus clusters? The overlap strongly depended on the size of the input network. While the overlap was notable for smaller networks (e.g., the embryonic gonad or brain), it was limited for larger networks. To illustrate the overlap between the different clusterings we calculated UPGMA trees based on the fraction of the intersection relative to the union (Jaccard index) of genes in MCL and MGclus clusters. The same was done for enriched GO terms. For the male adult gonad, a few MGclus and MCL clusters overlap to a high degree, but most of them do not have a counterpart with more than 30% overlap (Figure S2). On the other hand, for the male embryonic gonad, which had much fewer differential expressed genes, most of them find a counterpart with more than 60% overlap (Figure S3), indicating that these clusters are relatively reliable. Unsurprisingly, gene and GO term overlap trees were very similar.

**Figure 3:**
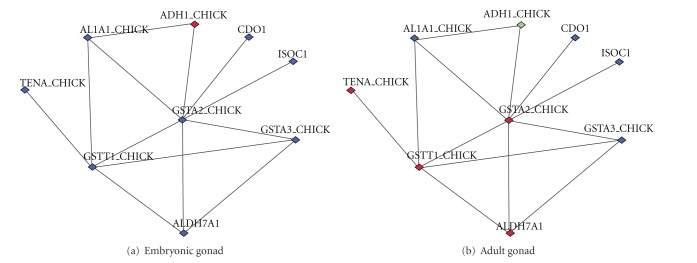
Example of sex bias switching between developmental stages. Shown is an MGclus cluster colored according to sex bias in the embryonic (a) and adult (b) gonads. Male-biased genes are shown in blue, female-biased in red, and unbiased in green.

One module identified from the embryonic gonad contained eight male-biased genes and one female-biased gene (Figure 3(a)). The female-biased gene was included because it was significantly enriched in connections to the male-biased genes. This module was functionally related to cell growth and development. It contained eight enzymes with biosynthetic functions and one extracellular matrix protein Tenascin (TENA_CHICK) which is important in tissue development. Two of the enzymes (AL1A1_CHICK, ADH1_CHICK) are important for retinoic acid (RA) synthesis [40]. RA is known to be crucial for embryonic development, growth, and reproduction. Four of the genes change sex bias and become female-biased in the adult gonad (Figure 3(b)), indicating that this module switches its function during development depending on the sex.

## 4. Discussion

By analyzing sex bias within the chicken gene network, we have been able to deduce several network properties pertaining to sexual dimorphism that gives new biological insights. Our analysis suggests that network hub genes tend not to be sex biased, although with some interesting exceptions. This suggests that most sex-biased genes tend to act within local network environments, and relatively few of them interact on a more global scale. This is consistent with recent studies that show that pleiotropy, as measured by expression breadth, tends to constrain the evolution of sex-biased expression [41, 42]. This analysis extends the measure of pleiotropy to network connectivity, with broadly consistent results.

We also investigated the propensity of sex-biased genes to form network modules in several ways. First, we noted that genes of the same sex bias tend to be more connected to each other than expected. Second, recently duplicated genes, which are similar in biochemical function, tend to have the same sex bias. Finally, a set of sex-biased modules were extracted from the network, and these showed unexpected functional homogeneity. These observations support a network structure that embodies sex-biased network modules. The implication of this is that the mechanisms underlying sex-specific development can be organized according to these modules, which simplifies the study and understanding of this complex system.

This work provides the first integrated, multidimensional analysis of the network structure underlying sex-biased gene expression and, as such, offers a more realistic link between sex-biased gene expression and sexually dimorphic phenotypes. Our analysis suggests, that rather than operating as distinct entities, genes of the same sex bias often group together in network modules, potentially due to shared regulatory elements or hierarchical pathway structures. This has several evolutionary genetic implications. First, it suggests that when many genes act in concert to encode sexually dimorphic phenotypes, they may be controlled by a shared regulatory apparatus. This collective regulatory control could then be exploited by emergent sexual dimorphisms, resulting in associated phenotypic differences [43]. It also suggests that single- or oligolocus models of sexual selection evolution (e.g., [44, 45]) are appropriate for some sexually dimorphic traits, even when transcriptome analysis reveals that gene expression of those phenotypes differs for many genes between the sexes. Although genes do not operate as independent units but are rather tethered in modules in a complex network of interactions, they however often work in concerted regulatory patterns. Therefore, our analysis somewhat paradoxically suggests that the control of complex sexual dimorphism may be ultimately attributable to relatively few key regulators.

Sex chromosomes often exhibit a nonrandom distribution of sex-biased genes associated with masculinizing or feminizing selection [46, 47]. Additionally, female heterogametic sex chromosomes, including those exhibited by birds, are also predicted to be particularly associated with the evolution of certain types of sexually selected traits [45, 48, 49]. Our analysis is consistent with these predictions. The crosstalk observed in the adult gonad between sex-biased genes on the Z chromosome and sex-biased genes on the autosomes suggests that the Z chromosome, which contains a relatively modest proportion of the total avian coding content, may play a disproportionately large role in the regulation of sex-biased genes. 

Previous work has shown a nonrandom distribution of sex-biased genes on the avian Z chromosome [50–52], with more male-biased and fewer female-biased genes on the Z chromosome than would be expected by chance alone. However this issue is complicated by the incomplete dosage compensation observed on the avian Z chromosome. Studies in a range of bird species have shown a persistent male bias on the Z chromosome due to the fact that males have two copies of every locus and females just one [34, 53, 54]. It has therefore been difficult to disentangle the effects of masculinizing selection for gene expression from incomplete dosage compensation [18]. Our analysis does not suffer from this type of conflation, as the crosstalk enrichment takes the relative abundances of different biases into account. This should minimize any effects of incomplete dosage compensation on our network.

In conclusion, our results suggest that network approaches to the study of sex-biased gene expression can offer new insights into the programming and genetic basis of sexual differentiation. Current transcriptome profiling produces massive datasets measuring relative gene expression, but this approach alone results in the false perception that each locus is independent of all others. Gene network approaches such as the one described here make it possible to consider a more multidimensional and integrated view of genome regulation which is particularly insightful for complex phenotypes.

## Supplementary Material

Supplementary Table S1: Microarray expression data sets that were used for the network training.
Supplementary Table S2: Top 20 of the most connected genes in the network. The level of differential expression is shown by FDR pvalues for the four tissue/stage conditions abbreviated as: G:gonad; B:brain; E:embryo; AD:adult.
Supplementary Table S3: Top 20 most sex-biased hub genes for each tissue/stage condition. Genes were first ranked according to sex bias or connectivity separately, and then re-ranked according to the sum of both ranks.
Supplementary Table S4: Total number of inparalog groups and the number of groups processed.
Supplementary Figure S1: The chicken network is scale-free, manifested by a power-law frequency distribution of node degrees, which becomes linear in a log-log plot.
Supplementary Figure S2: Relationship between MCL and MGclus clusters for the male adult gonad in terms of a) genes and b) enriched GO terms.
Supplementary Figure S3: Relationship between the MCL and MGclus clusters for the male embryonic gonad in terms of a) genes and b) enriched GO terms.

## Figures and Tables

**Table 1:** Number of links (first number) and unique genes (second number) at different FBS (final Bayesian score) cutoffs in FunCoup, where *c* is the corresponding confidence value of functional coupling.

	Metabolic	Signaling	Combined	Total
FBS > 3 (*c* > 0.02)	1375931/10555	601101/10569	1152763/10549	1809810/11311
FBS > 5.9 (*c* > 0.25)	171490/5520	33934/4861	124616/5383	199120/6748
FBS > 7 (*c* > 0.5)	89818/4132	13401/2990	62707/3885	100887/4902
FBS > 8 (*c* > 0.75)	52285/3175	6365/1869	35821/2930	57690/3673

**Table 2:** Number of sex-biased and unbiased genes separated by the cut-off FDR < 0.1 according to MWT.

	Male	Female	Unbiased
Embryonic gonad	1304	1128	3244
Adult gonad	1934	2693	1049
Embryonic brain	0	2	5675
Adult brain	87	92	5497

**Table 3:** Sex-biased genes tend not to be hubs. This is evidenced by Spearman's correlation coefficient between differential expression (measured as MWT FDR) and network connectivity which was significantly positive in most cases. Sex-biased genes were separated from unbiased genes using a cutoff of FDR = 0.1. The first number is the correlation, and in brackets is the corresponding *P* value. Significant correlations (*P* < 0.01) are marked in bold.

	Male	Female	Unbiased
Embryonic, gonad	**0.12** **(**7.98**e** − 06**)**	**0.16** **(**1.55**e** − 07**)**	−0.03 (0.12)
Adult, gonad	−0.04 (0.10)	**0.06** **(**9.91**e** − 04**)**	−0.003 (0.93)
Adult, brain	0.12 (0.29)	−0.06 (0.60)	0.03 (0.02)

**Table 4:** Results of the inparalog group analysis showing the number of groups in the various categories. In total we found 69 groups with at least two inparalogs in chicken. However, only 59 could be processed since expression data were not available for all genes in ten of the groups. The number in brackets in the mixed cluster field is the number of groups that contain both male- and female-biased genes. A significant difference between the observed number of inparalogs and what is expected by chance is indicated by *(*P* < 0.05), + indicates a number higher than expected by chance, and − a number lower than expected.

	Gonad adult	Gonad embryo	Brain adult
All-male	15+*	7+*	0
All-female	18+*	8+*	0
All-unbiased	6+*	25	58+
Mixed	20 (5)−*	19 (1)−*	1 (0)−

**Table 5:** Number of MGclus and MCL clusters, number of clusters with significant GO term enrichment, and the level of significant GO term enrichment compared to random. A *z*-score above 2 corresponds to a significance level of *P* < 0.05.

	Clusters	Significant	Avg. sig. terms	Random avg. sig. terms
MGclus				

Gonad embryo male	6	6	43.50	18.72
Gonad adult male	24	24	20.58	9.56
Brain adult male	3	3	16.67	13.01
Gonad embryo female	6	6	31.17	16.09
Gonad adult female	22	22	62.23	23.58
Brain adult female	3	3	25.67	20.94

MCL				

Gonad embryo male	5	5	30.80	16.87
Gonad adult male	31	29	18.52	6.92
Brain adult male	6	6	9.00	6.94
Gonad embryo female	3	3	16.67	10.85
Gonad adult female	64	64	27.97	10.62
Brain adult female	3	3	24.33	16.42
