# Supplementary material for: Network Analysis of Functional Genomics Data: Application to Avian Sex-Biased Gene Expression
Source: ScientificWorldJournal. 2012 Dec 23;2012:130491. doi: 10.1100/2012/130491 (PMC3540752; doi:10.1100/2012/130491)

Table S1: Microarray expression data sets that were used for the network training.

GEO accession number	Number of Samples	Platform
GSE9745	80	GPL6049 Arizona Gallus gallus 20.7K
GSE8812	24	GPL1731 DEL-MAR 14K Integrated Systems
GSE7254	40	GPL1731 DEL-MAR 14K Integrated Systems
GSE5205	23	GPL1731 DEL-MAR 14K Integrated Systems
GSE5067	48	GPL1731 DEL-MAR 14K Integrated Systems
GSE10052	8	GPL1731 DEL-MAR 14K Integrated Systems
GSE10538	24	GPL3213 Affymetrix GeneChip Chicken Array

Table S2: Top 20 of the most connected genes in the network. The level of differential expression is shown by FDR p-values for the four tissue/stage conditions abbreviated as: G:gonad; B:brain; E:embryo; AD:adult.

Gene	Connectivity	FDR G/E	FDR G/AD	FDR B/E	FDR B/AD
ENSGALG00000002569	1316	4.00E-03	1.90E-05	9.94E-01	4.13E-01
GTP-binding nuclear protein Ran (GTPase Ran) (Ras-like protein TC4).					
ENSGALG00000006512	1077	9.52E-01	3.47E-01	9.95E-01	9.29E-01
heat shock cognate 70					
ENSGALG00000005931	900	4.24E-01	9.47E-05	9.94E-01	2.84E-01
RuvB-like 1					
ENSGALG00000004707	895	2.00E-02	1.00E-02	9.94E-01	9.33E-01
Exportin-2 (Exp2)(Importin-alpha re-exporter)(Chromosome segregation 1-like protein)(Cellular apoptosis susceptibility protein)					
ENSGALG00000008812	882	3.50E-01	5.40E-02	9.94E-01	6.38E-01
chaperonin containing TCP1, subunit 4 (delta)					
ENSGALG00000014395	849	6.30E-01	3.68E-04	9.98E-01	7.63E-01
DEAH (Asp-Glu-Ala-His) box polypeptide 15					
ENSGALG00000014450	849	3.68E-01	2.16E-01	9.98E-01	8.91E-01
poly(A) binding protein, cytoplasmic 1					
ENSGALG00000007711	848	4.94E-01	5.00E-03	9.94E-01	8.03E-01
ribosomal protein L4					
ENSGALG00000013041	828	4.30E-02	3.30E-01	9.96E-01	7.22E-01
chaperonin containing TCP1, subunit 5 (epsilon)					
ENSGALG00000009975	820	6.98E-01	8.54E-05	1.00E+00	8.93E-01
chaperonin containing TCP1, subunit 2 (beta)					
ENSGALG00000003046	817	1.46E-01	6.04E-01	9.95E-01	9.43E-01
Proteasome subunit alpha type-4 (EC 3.4.25.1)(Proteasome component C9)(Macropain subunit C9)(Multicatalytic endopeptidase complex subunit C9)(Proteasome subunit L)					
ENSGALG00000011174	811	3.50E-02	1.32E-01	9.94E-01	8.89E-01
proteasome (prosome, macropain) subunit, beta type, 1					
ENSGALG00000004269	794	8.50E-02	2.72E-04	9.94E-01	9.41E-01
PREDICTED: Gallus gallus similar to Prt1 homolog (LOC769783), mRNA.					
ENSGALG00000000454	793	3.00E-03	1.33E-01	9.94E-01	5.46E-01
Importin subunit beta-1 (Karyopherin subunit beta-1)(Nuclear factor P97)(Importin-90)					
ENSGALG00000006910	785	2.44E-01	5.90E-02	9.96E-01	8.36E-01
inosine monophosphate dehydrogenase 2					
ENSGALG00000009913	776	8.30E-02	1.40E-02	9.94E-01	9.93E-01
Hypothetical protein.					

ENSGALG00000009325	750	3.00E-03	1.87E-05	9.94E-01	2.62E-01
GTP-binding protein PTD004					
ENSGALG00000000065	749	na	na	na	na
proteasome alpha 5 subunit					
ENSGALG00000010060	738	5.00E-03	2.30E-04	9.94E-01	8.26E-01
Proteasome subunit alpha type-6 (EC 3.4.25.1)(Proteasome iota chain)(Macropain iota chain)(Multicatalytic endopeptidase complex iota chain)(27 kDa prosomal protein)(PROS-27)(p27K)					
ENSGALG00000008684	728	2.29E-01	8.85E-05	9.94E-01	7.45E-01
Eukaryotic initiation factor 4A-II (EC 3.6.1.-) (ATP-dependent RNA helicase eIF4A-2) (eIF4A-II) (eIF-4A-II).					

Table S3: Top 20 most sex-biased hub genes for each tissue/stage condition. Genes were first ranked according to sex bias or connectivity separately, and then re-ranked according to the sum of both ranks.

Embryonic gonad			
	**Gene**	**Connectivity**	**log2 m/f**
1	ENSGALG00000010439, TUBA3E, tubulin, alpha 3e	426	-2.40
2	ENSGALG00000006885, RASH, GTPase HRas	458	1.13
3	ENSGALG00000010235, DDX19B, DEAD (Asp-Glu-Ala-As) box polypeptide 19	498	0.93
4	ENSGALG00000003914, CALR3, Calreticulin-3	249	-2.92
5	ENSGALG00000008462, CDK3, Cell division protein kinase 3	189	-3.66
6	ENSGALG00000000086, TIMM17A, translocase of inner mitochondrial membrane 17 homolog A	197	-2.39
7	ENSGALG00000006110, PLK1, polo-like kinase 1	336	1.10
8	ENSGALG00000009844, ACTC1, actin, alpha, cardiac muscle 1	197	3.22
9	ENSGALG00000016676, MCM3, minichromosome maintenance complex component 3	318	1.00
10	ENSGALG00000004325, P4HA1, prolyl 4-hydroxylase, alpha polypeptide I	156	1.26
11	ENSGALG00000001992, PKM2, pyruvate kinase, muscle	468	0.72
12	ENSGALG00000002797, Q5ZIZ0, 6-phosphogluconate dehydrogenase, decarboxylating	216	1.01
13	ENSGALG00000007611, RPL35A, ribosomal protein L35a	376	0.91
14	ENSGALG00000009312, RPL22L1, ribosomal protein L22-like 1	132	1.26
15	ENSGALG00000006520, MYH11, myosin, heavy chain 11, smooth muscle	67	3.58
16	ENSGALG00000000681, PAK1, p21 protein (Cdc42/Rac)-activated kinase 1	47	2.75
17	ENSGALG00000014331, FKBP4, FK506 binding protein 4, 59kDa	272	0.81
18	ENSGALG00000007719, SHANK2, SH3 and multiple ankyrin repeat domains 2	76	2.95
19	ENSGALG00000007662, SPO11, meiotic protein covalently bound to DSB	37	-5.22
20	ENSGALG00000010255, NASP, nuclear autoantigenic sperm protein (histone-binding)	97	1.59
**Adult gonad**			
	**Gene**	**Connectivity**	**log2 m/f**
1	ENSGALG00000016887, IPO5, importin 5	654	3.26
2	ENSGALG00000010439, TUBA3E, tubulin, alpha 3e	426	6.45
3	ENSGALG00000004809, PPIF, peptidylprolyl isomerase F	369	-3.92
4	ENSGALG00000012159, ACTR3, ARP3 actin-related protein 3 homolog	557	-2.79
5	ENSGALG00000008462, CDK3, Cell division protein kinase 3	189	5.02
6	ENSGALG00000013056, TUBA4A, tubulin, alpha 4a	159	9.57
7	ENSGALG00000008163, PSME4, proteasome (prosome, macropain) activator subunit 4	252	3.59
8	ENSGALG00000016410, EIF1AY, eukaryotic translation initiation factor 1A, Y-linked	178	5.71
9	ENSGALG00000011738, ARHGDIB, Rho GDP dissociation inhibitor (GDI) beta	167	-4.53
10	ENSGALG00000013045, TUBAL2, TUBA8, testis-specific alpha-tubulin	94	9.57
11	ENSGALG00000012834, AKR1D1, aldo-keto reductase family 1, member D1 (delta 4-3-ketosteroid-5-beta-reductase)	98	-7.69
12	ENSGALG00000003998, RAB8A, member RAS oncogene family	326	-2.99
13	ENSGALG00000010207, LARP1B, La ribonucleoprotein domain family, member 1B	136	4.68
14	ENSGALG00000007307, USP7, ubiquitin specific peptidase 7	152	4.56
15	ENSGALG00000006799, AP2A2 ,adaptor-related protein complex 2, alpha 2 subunit	250	-3.28
16	ENSGALG00000007037, PP6R3, Serine/threonine-protein phosphatase 6 regulatory subunit 3	119	4.11
17	ENSGALG00000010235, DDX19B, DEAD (Asp-Glu-Ala-Asp) box polypeptide 19B	498	-2.27
18	ENSGALG00000009641, COL1A2, collagen, type I, alpha 2	105	-5.13
19	ENSGALG00000023207, H2B1, Histone H2B 1/2/3/4/6	149	4.04
20	ENSGALG00000023210, H2B-VII, histone H2B 1/2/3/4/6	148	4.04

Adult brain			
	**Gene**	**Connectivity**	**log2 m/f**
1	ENSGALG00000009250, HNRNPA3, heterogeneous nuclear ribonucleoprotein A3	522	-0.75
2	ENSGALG00000008348, PDIA3, protein disulfide isomerase family A, member 3	412	0.59
3	ENSGALG00000001103, PSMB7, proteasome (prosome, macropain) subunit, beta type, 7	618	-0.70
4	ENSGALG00000007615, RAB11A, member RAS oncogene family	438	0.46
5	ENSGALG00000013990, RPS12, ribosomal protein S12	325	-0.51
6	ENSGALG00000005694, GARS, glycyl-tRNA synthetase	319	-0.56
7	ENSGALG00000012233, EIF4E, eukaryotic translation initiation factor 4E	535	0.36
8	ENSGALG00000010238, NAP1L1, nucleosome assembly protein 1-like 1	290	-0.53
9	ENSGALG00000009615, RRP15, ribosomal RNA processing 15	287	-0.45
10	ENSGALG00000003985, TARSL2, threonyl-tRNA synthetase-like 2	194	0.63
11	ENSGALG00000015450, RAB2A, member RAS oncogene family	404	0.43
12	ENSGALG00000004133, SRSF10, serine/arginine-rich splicing factor 10	157	-0.58
13	ENSGALG00000012402, CUL1, cullin 1	211	0.55
14	ENSGALG00000015043, RPF2, ribosome production factor 2	331	0.34
15	ENSGALG00000009964, YEATS4, YEATS domain containing 4	273	0.41
16	ENSGALG00000007931, DLD, dihydrolipoamide dehydrogenase	342	0.33
17	ENSGALG00000008095, KIF23, kinesin family member 23	176	0.70
18	ENSGALG00000012655, POLR3B, polymerase (RNA) III (DNA directed) polypeptide B	232	0.45
19	ENSGALG00000017254 DLG2, discs, large homolog 2 (Drosophila)	96	0.83
20	ENSGALG00000011330, DYNC1H1, dynein, cytoplasmic 1, heavy chain 1	176	0.53

Table S4: Total number of inparalog groups and the number of groups processed. Note that not all groups could be processed since expression data were in some cases not available for all genes in a cluster. Expected number of inparalog groups from random sampling, and the significance of the observed number of inparalog groups in different categories. A z-score of 2 means 95% confidence interval.

	**gonad adult**	**gonad embryo**	**brain adult**
**#inparalog groups**	133	133	133
**#processed groups**	69	69	69
**#incomplete groups**	10	10	10
**#avg. male groups (sd)**	6.77 (2.44)	1.94 (1.33)	0.008 (0.09)
**#avg. female groups (sd)**	7.865 (2.59)	2.00 (1.35)	0.011 (0.10)
**#avg. unbiased groups (sd)**	2.637 (1.59)	18.52 (3.68)	55.19 (1.85)
**#avg. mixed groups (sd)**	41.728 (3.40)	36.536 (3.75)	3.791 (1.84)
**z-score male**	3.38	3.81	-0.09
**z-score female**	3.92	4.46	-0.11
**z-score unbiased**	2.12	1.76	1.52
**z-score mixed**	-6.39	-4.68	-1.51
**fraction lower than observed male**	0.997	0.997	0.00
**fraction lower than observed female**	0.999	0.999	0.00
**fraction lower than observed unbiased**	0.961	0.948	0.90
**fraction lower than observed mixed**	0.000	0.000	0.02

Figure S1: The chicken network is scale-free, manifested by a power-law frequency distribution of node degrees, which becomes linear in a log-log plot.

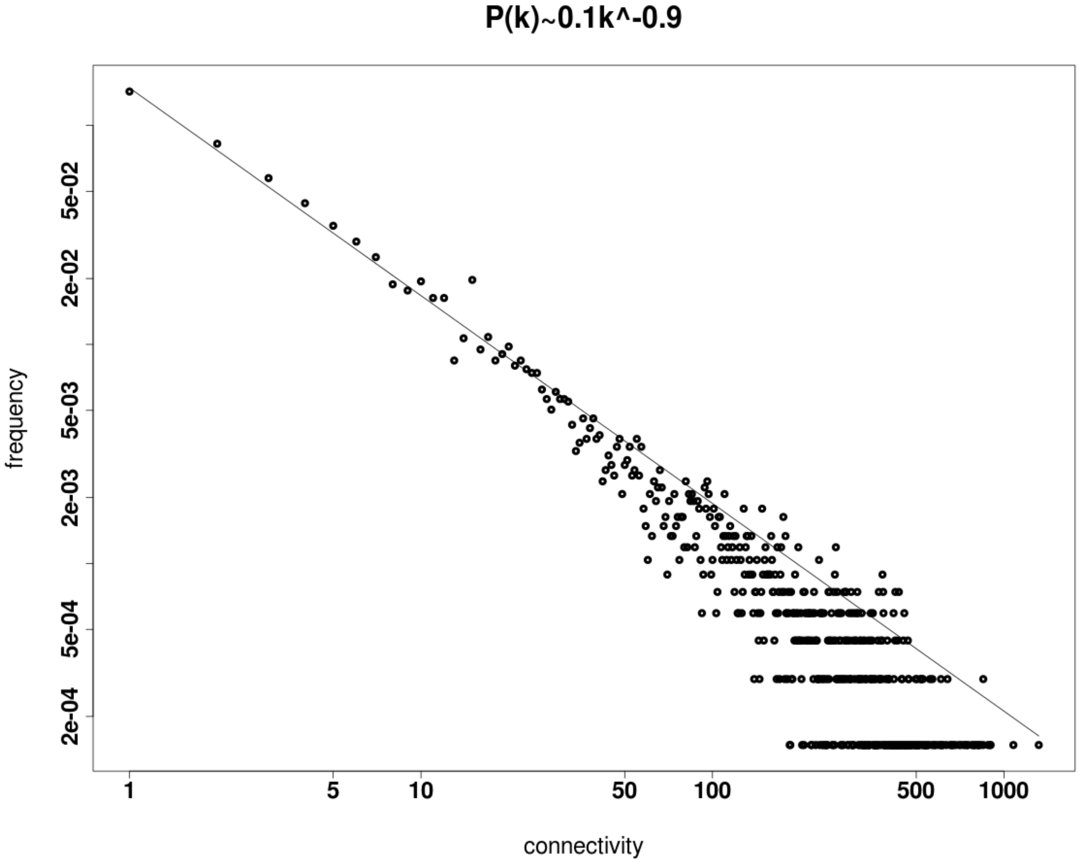

Figure S2. Relationship between MCL and MGclus clusters for the male adult gonad in terms of a) genes and b) enriched GO terms.

a) Shared genes **b) Shared enriched GO terms**

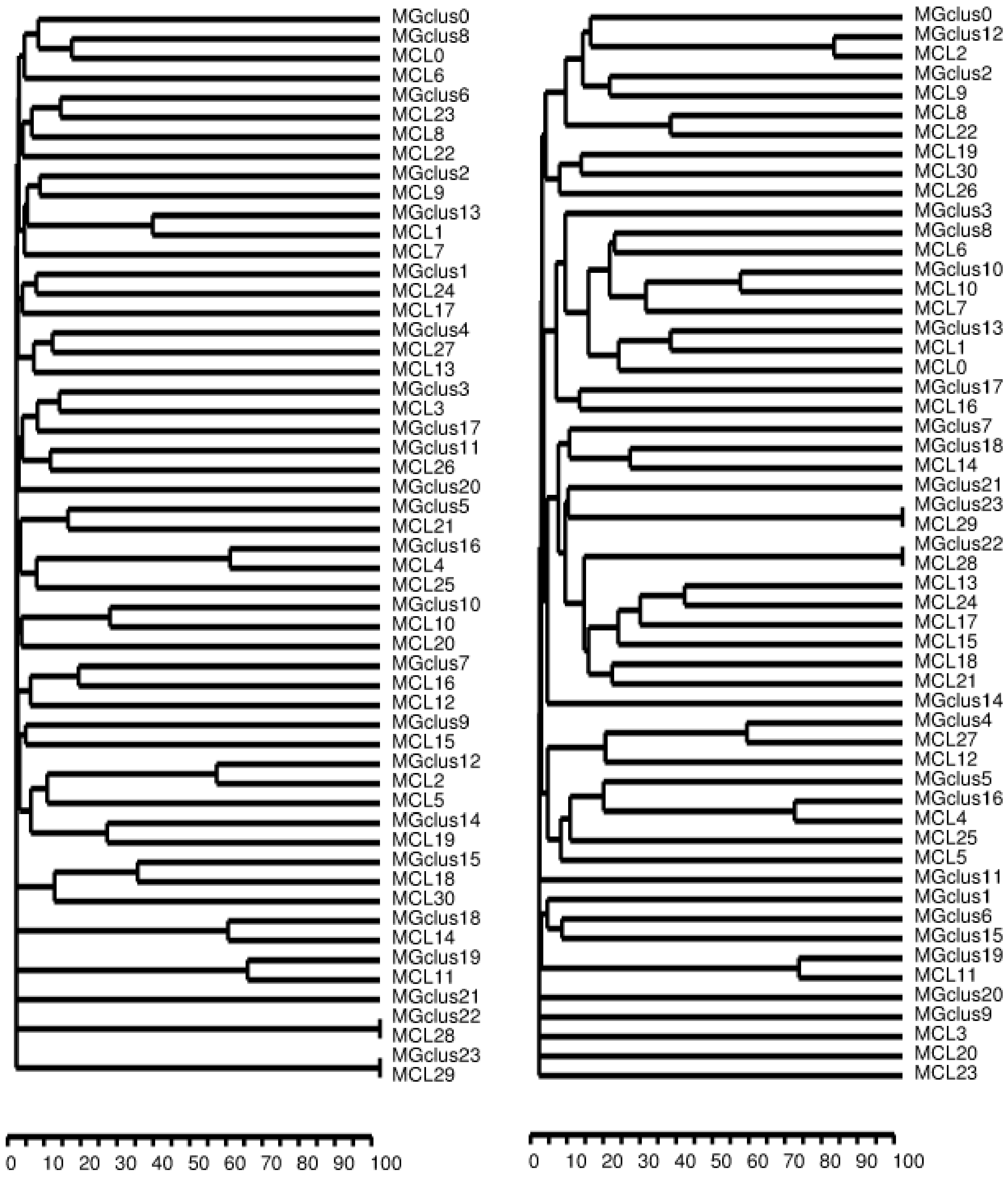

Figure S3: Relationship between the MCL and MGclus clusters for the male embryonic gonad in terms of a) genes and b) enriched GO terms.

a) Shared genes

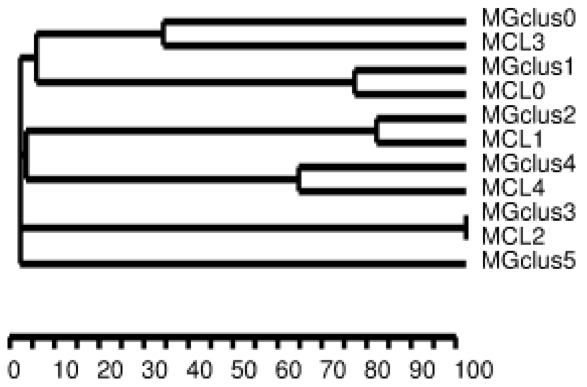

b) Shared enriched GO terms

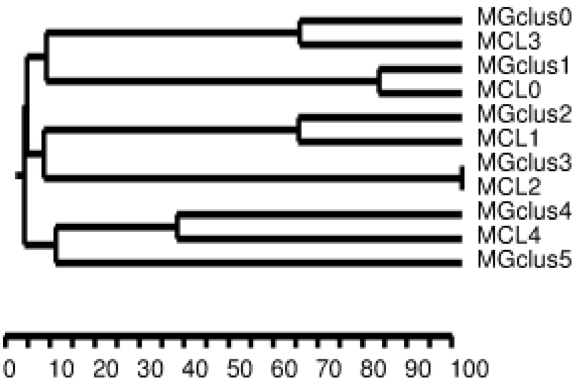

Supplement: Supplementary file 1 — Supplementary Table S1: Microarray expression data sets that were used for the network training. Supplementary Table S2: Top 20 of the most connected genes in the network. The level of differential expression is shown by FDR pvalues for the four tissue/stage conditions abbreviated as: G:gonad; B:brain; E:embryo; AD:adult. Supplementary Table S3: Top 20 most sex-biased hub genes for each tissue/stage condition. Genes were first ranked according to sex bias or connectivity separately, and then re-ranked according to the sum of both ranks. Supplementary Table S4: Total number of inparalog groups and the number of groups processed. Supplementary Figure S1: The chicken network is scale-free, manifested by a power-law frequency distribution of node degrees, which becomes linear in a log-log plot. Supplementary Figure S2: Relationship between MCL and MGclus clusters for the male adult gonad in terms of a) genes and b) enriched GO terms. Supplementary Figure S3: Relationship between the MCL and MGclus clusters for the male embryonic gonad in terms of a) genes and b) enriched GO terms. [file 130491.f1.pdf]
